# Supplementary material for: Diet Quality, Health, and Wellbeing within the Irish Homeless Sector: A Qualitative Exploration
Source: Int J Environ Res Public Health. 2022 Nov 30;19(23):15976. doi: 10.3390/ijerph192315976 (PMC9738806; doi:10.3390/ijerph192315976)
Supplement: Supplementary file 1 [file ijerph-19-15976-s001.zip › ijerph-2014639-supplementary.pdf]

	“Before I became homeless, you know like, I’d have control over what I eat like, you know. And em…and I love food, this is the thing like. I really love food and I love cooking; you know and that’s all gone now, you know. It’s eh…the last half a year, that’s gone, you know” [Man, service user]<br><br>“I think about the food I…the variety of food I used to have like and, and it can be quite depressing, sometimes, frankly like…. kind of thinking about the variety of food I used to have like that I don’t have any more like, you know, like. Because I love food” [Man, service user]<br><br>“We can’t…. say if somebody had a barbecue and came back in with or somebody had a family gathering and they have a rake of sandwiches leftover and they come in here to us, we can’t use them because they’re potentially ha-, they’re contaminated. So, there’s only limited sort of stuff. What…what we do with some type of donated food is rather…we accept it, but we don’t use it, we have to discard it” [Man, service user]<br><br>“I think it’s just even for mum being able to provide for her children. That’s a massive thing. All the mothers say, “I just want to cook. My child comes home from school, I can’t have a dinner ready for them. My child comes home from school, and I have to kind of organise everything else so I can bring them out even to have a meal or something.”” [Woman, service provider]
	“Most days like McDonald’s, I bring the kids to McDonald’s” [Woman, service user]<br><br>“Em….it would be cafés, that’s where we got our pancake addiction as I call it” [Woman, service user]<br><br>“Well in the morning, you have cereal, boiled eggs, toasts, fruit – bananas whatever, you know. You have your choice. Lunch - soup, salad, sandwiches. Dinner – you have your potatoes, two veg, different meats, all very well prepared. And then 8 o’ clock in the evening you get more sandwiches if you want” [Man, service user]<br><br>“There’s a variety of food so every day there’s a choice. It could be chicken or beef…em or it could be fish and chicken, or you know, it could a rice dish or a pasta dish. So, every day there’s, there’s a choice which is the right way to have it” [Man, service provider]<br><br>“So, what most people do when they come into here at lunch time, they’ll have…they’ll get a selection, a bowl of soup. We give them what they ask for, if they say a bowl of soup, we give them a bowl of soup… we get our sliced bread, there’s a white bread and a wholemeal bread… I suppose the most popular one is the ham and cheese which is on brown bread” [Man, service provider]

	"No, see that's it, I don't have nothing, I've a kettle in the room, that's it" [Man, service user]<br><br>"Now we're back up in the B&B. Em but there's no cooking facilities, but they have microwave and that so… There's a fridge we can store our stuff but we're sharing it with other people, you know… but you're thinking then will somebody else take our food?" {Woman, service user]<br><br>"I don't really have an option to like to prepare my own meals. I'd love the chance to do that myself like but uh…they don't really have anything other than boiling water to offer" [Man, service user]<br><br>"In a B&B there is either a fridge in your room or there is a communal fridge that can be used and cupboards…cupboards not so much, fridge yes. There's always shelving obviously in a room so that turns into a cupboard…And that's [fridges] just for storing kind of milk and bits and pieces but yoghurts maybe but I find that even if someone has a fridge, it's for the like minimal. They don't seem to store anything cause there's no point in storing vegetables cause you can't cook them… There's no point in storing meat, you can't cook it" [Woman, service provider]
	"I don't eat healthy, love to but I can't…. don't eat healthy. Em…. let me see, eh…. like that because there's no proper food I kind of more turn to fizzy drinks to give me some energy, d'you know, because I do be feeling so weak" [Woman, service user]<br><br>"You know, you have that fall from grace and you have that type of shame where it's open knowledge…well it's not open know-, its whispered you're homeless and you're on pull full display as such with your little old lady shopping trolley and ugh…. it's just psychologically hard work" [Woman, service user]<br><br>"Cause when I had the flu for the, you know, three times I had the flu and, on each occasion, I was about two weeks where I could hardly even leave the B&B. So, I could hardly even buy lunch" [Woman, service user]<br><br>"We feel, you know, that if we can get that right, we can get the environment right, so a combination of good food, clean environment and a structured environment, well then, you're halfway there, you know, giving people some sort of sense of being" [Man, service provider]<br><br>"One would think while they're in…in that form of a say accommodation that their nutrition would improve but we don't necessarily see that in bloods. So, we might do a blood when they come in and a few weeks later we might just check it again if it was a bit off and it might often be worse" [Man, service provider]

Table S1. Sample of quotations to represent each theme
